# Sphingosine 1-phosphate receptor 2 in keratinocytes plays a key role in reducing inflammation in psoriasis

**DOI:** 10.3389/fimmu.2024.1469829

**Published:** 2024-09-26

**Authors:** Kana Masuda-Kuroki, Shahrzad Alimohammadi, Samantha Lowry, Anna Di Nardo

**Affiliations:** Department of Dermatology, School of Medicine, University of California San Diego, La Jolla, CA, United States

**Keywords:** keratinocyte biology, psoriasis, sphingosine 1-phosphate, sphingosine 1-phosphate receptor 2, Th 17 cells

## Abstract

**Background:**

Psoriasis is an inflammatory skin condition where immune cells play a significant role. The importance of the cross-talk between keratinocytes and immune cells in the pathogenesis of psoriasis has recently been reaffirmed. Recent studies have found that several S1PR functional antagonists, other than S1PR2, are effective in improving psoriasis. This study aims to investigate the role of S1PR2 in psoriasis, that has not been investigated before.

**Methods:**

Spatial transcriptomics, RT-qPCR, and flow cytometry were used to map the immune cell landscape and its association with metabolic pathways in an imiquimod (IMQ)-induced psoriasis-like inflammation in *S1pr2^fl/fl^ K14-Cre* mice that could not sense sphingosine-1-phosphate (S1P) in the epidermis through the S1PR2 receptor.

**Results:**

Our analysis suggests that S1PR2 in keratinocytes plays a major role in psoriasis-like inflammation compared to other S1PRs. It acts as a down-regulator, inhibiting the recruitment of Th17 cells into the skin. In IMQ-induced psoriasis skin, both *S1pr2^-/-^* and *S1pr2^fl/fl^ K14-Cre* mice showed higher expressions of proinflammatory cytokines such as TNF-α, IL-17A, and IL-1β together with higher expressions of MyD88/NF-κB pathway compared to the wild-type mice. Remarkably, in IMQ-treated mice, the deletion of *S1pr2* in keratinocytes only resulted in a larger population of Th17 cells in skin-draining lymph nodes. Other S1PR modulators did not improve the worsening of psoriasis-like inflammation caused by S1PR2 deficiency in keratinocytes.

**Conclusion:**

This study reaches two main conclusions: signals from keratinocytes play a central role in creating an immune environment that promotes the development of psoriasis, and stimulating S1PR2, instead of suppressing it, represents a potential therapeutic approach for psoriasis.

## 1 Introduction

Psoriasis is a chronic inflammatory skin disease characterized by scaly and elevated erythema, which involves numerous cell types in its inflammatory cascades. Recent studies on the pathogenesis of psoriasis have focused on immune cells and revealed that activated dendritic cells (DCs) promote helper T cell differentiation into Th1 and Th17 cells, which secrete cytokines stimulating epidermal keratinocytes to form hyperproliferation (1). On the other hand, keratinocytes respond to stimuli by secreting antimicrobial peptides, cytokines, and chemokines, further promoting the activation of T cells, DCs, and neutrophils to circulate an inflammatory loop to evoke a chronic inflammation (2, 3). Therefore, keratinocytes are considered a key factor in the inflammatory loop not only as an executor but also as a trigger for immune responses in psoriasis (4).

Keratinocytes constitute the outermost layer of the skin and are surrounded by an intercellular lipid matrix composed of ceramides, cholesterol, and fatty acids (5). Sphingosine 1-phosphate (S1P), one of the bioactive sphingolipid mediators, is metabolically generated from ceramide (6). S1P regulates cellular activities, including cell proliferation and immune responses, by binding to the G-protein-coupled receptor S1PR1-5 (7).

Recently, several groups have found that the levels of sphingosine, S1P, and ceramide in psoriasis lesional skin are significantly higher than in healthy skin in humans (8, 9). Moreover, lipidomic profiling has revealed that the skin lesions in imiquimod (IMQ)-induced psoriasis mouse models have higher ceramides than those in the control mouse skin (10). In addition, some research groups treated psoriasis mice with S1P or S1PR modulators except for S1PR2 (11, 12). These studies confirmed that Sphingolipids and their signaling, except S1PR2, promote the pathogenesis of psoriasis. However, it has not been directly confirmed whether direct inhibition or promotion of S1PR2 alters psoriasis inflammation. Thus, it was still unclear whether S1PR2 has any role in psoriasis. Since previous studies found that S1PR2 is involved in proinflammatory cytokine production in the epidermis as a response to various external stimuli (13) and has a role in regulating keratinocyte proliferation (14), we hypothesized that S1PR2 plays a vital role in the pathogenesis of psoriasis.

This report demonstrates the involvement of S1PR2 in keratinocytes in regulating psoriasis-like inflammation. This study investigates induced psoriasis inflammation in S1PR2-knockout mice, keratinocyte-specific S1PR2 conditional knockout mice, and S1PR2-blocked human keratinocytes. Our study found that the S1PR2 deficiency in keratinocytes activates the myeloid differentiation primary response gene 88 (MyD88)/nuclear factor-kappa B (NF-κB) pathway, leading to Th17 recruiting into the skin.

Here, we present compelling evidence that keratinocytes play a central role in initiating psoriasis inflammation and regulating other cells through S1PR2 signaling. This pathway may offer a novel treatment for psoriasis.

## 2 Materials and methods

### 2.1 Mice

Balb/c mice, that is *S1pr2^+/+^* were purchased from Jackson Lab (Bar Harbor, ME). *S1pr2^-/-^* mice were provided by Dr. Chun’s laboratory (15). *S1pr2^fl/fl^* mice were gifted from Dr. Hla’s laboratory at Boston Children’s Hospital (MA, USA). *K14-Cre*-transgenic mice were gifted from Dr. Gallo’s laboratory at the University of California San Diego (CA, USA). Epidermis-specific S1PR2 knockout mice (*S1pr2^fl/fl^ K14-Cre*) were generated by crossing *S1pr2^fl/fl^* mice with *K14-Cre*-transgenic mice as described previously (16). All mice were housed in the laboratory animal facility at the University of California San Diego in individual ventilated plastic cages and were provided food and water *ad libitum* under temperature-controlled conditions with a 12 h light/dark cycles. All experiments were performed with 3 times with at least 3 mice in each group each time. For a total of at least 9 mice were used for each group in the experiment. All animal protocols were reviewed and approved by University of California San Diego (IACUC approval number: s10288).

### 2.2 Psoriasis mouse models

Mice at 8 weeks old of *S1pr2^+/+^*, *S1pr2^-/-^*, *S1pr2^fl/fl^*, and *S1pr2^fl/fl^ K14-Cre* were depilated on the back skin 1 day before the treatment. After depilation, mice received a daily topical dose of 62.5 mg of commercially available IMQ cream (5%) (Catalog No. 51672414506, Taro Pharmaceutical Industries, Hawthorne, NY) for 7 days on the depilated back skin as described previously (17). Over 7 days, one group of mice received 10 μg of FTY720 dissolved in 80μL of ethanol 1 hour before administration of IMQ. FTY720 dosage and administration timing were per previous papers (18, 19). Skin samples and axillary lymph node (LN)s were collected for further analysis 24 hours after the last IMQ application. The severity of erythema and scale was evaluated using an objective scoring system from 0 to 4 (0, none; 1, slight; 2, moderate; 3, marked; and 4, very marked) as described previously (20). Axillary LNs were measured using Kynup Digital Caliper (Deqing Liang Feng Electronic & Technology Co., Ltd., Zhejiang, China), and weighed by Mettler Toledo Analytical Balance MS104TS/00 (Catalog No. 30133522, Mettler Toledo, Columbus, OH).

### 2.3 Cell culture

Primary normal human epidermal keratinocytes NHEKs (Catalog No. C0015C, Thermo Fisher Scientific, Waltham, MA) were cultured in EpiLife Medium with 60 mM calcium (Catalog No. MEPI500CA, Thermo Fisher Scientific, Waltham, MA) supplemented with Human Keratinocyte Growth Supplement (Catalog No. S0015, Thermo Fisher Scientific, Waltham, MA) at 37 °C in 5% CO_2_. Subconfluent cells were seeded in 6-well plates and grown to 80% confluence before treatment.

### 2.4 Chemical S1PR2 block and IMQ treatment on NHEKs

NHEKs cultured in 6-well plates were incubated with phosphate-buffered saline (PBS) or 10 μM of JTE013 (Catalog No. 10009458, Cayman Chemical Company, Ann Arbor, MI) at 37°C in 5% CO_2_ for 2 hours (13), then incubated with PBS for 24 hours or 100 μM of IMQ (Catalog No. 99011-78-6, InvivoGen, San Diego, CA) for 2, 5, or 24 hours at 37°C in 5% CO_2_ (21, 22).

### 2.5 Real-time reverse transcription-quantitative polymerase chain reaction

Total RNAs from mouse dorsal epidermis and NHEKs were obtained by Quick RNA Miniprep Kit (Catalog No. R1055, Zymo Research, Irvine, CA). cDNA conversion from RNA and real-time RT-qPCR were performed according to the previous report (16). The primers and the probes used for real-time RT-qPCR are listed in **Supplementary Tables S1, S2**, respectively. The volume of RNA and cDNA was measured by NanoDrop 2000 Spectrophotometer (Catalog No. ND2000CLAPTOP, Thermo Scientific, Waltham, MA). The expression of target genes was normalized to glyceraldehyde-3-phosphate dehydrogenase (GAPDH) expression and analyzed by the 2^– ΔΔct^ method.

### 2.6 Histology and immunofluorescence staining

Formalin-fixed and paraffin-embedded (FFPE) mouse skin samples were sectioned, and HE stained at the University of California San Diego Biorepository and Tissue and Technology Shared Resource. For immunofluorescence staining of FFPE sections, unstained sections were deparaffinized, rehydrated, and allowed to reach boiling temperature and maintained at a sub-boiling temperature for 10 minutes with antigen retrieval solution (Catalog No. 00495558, Invitrogen, Waltham, MA). After antigen retrieval, immunofluorescent staining was performed using the primary and fluorochrome-conjugated secondary antibodies listed in **Supplementary Table S3**. Nuclei were counterstained with 4’,6-diamidino-2-phenylindole (DAPI) and slides were mounted with Prolong Gold Anti-Fade Reagent with DAPI (Catalog No. 8961, Cell Signaling Technology, Danvers, MA).

For immunofluorescence staining of NHEKs, cells were cultured on the cover glass in 10-cm petri dish for 24 hours and fixed with 4% paraformaldehyde. Fixed cells were stained with primary and fluorochrome-conjugated secondary antibody listed in **Supplementary Table S3**. For negative control, fixed cells were stained only with fluorochrome-conjugated secondary antibody. Nuclei were counterstained with DAPI with Prolong Gold Anti-Fade Reagent with DAPI (Catalog No. 8961, Cell Signaling Technology, Danvers, MA). Fluorescence images were obtained with a fluorescence microscope EVOS M5000 (Catalog No. AMF5000, Invitrogen, Waltham, MA).

### 2.7 Flow cytometry

Axillary LNs were extracted from mice, and LN cells were isolated for flow cytometry analysis. Surface staining was performed for 20 minutes at 4°C with fluorochrome-conjugated antibodies. Fc receptors were blocked with BD Pharmingen Purified Rat Anti-Mouse CD16/CD32 (Mouse BD Fc Block) (Catalog No. AB_394656, BD Biosciences, Franklin Lakes, NJ) diluted 1:100 before staining. For intracellular staining, the cells were fixed and permeabilized using BioLegend Cyto-Fast Fix/Perm Buffer Set (Catalog No. 426803, BioLegend, San Diego, CA), then stained with fluorochrome-conjugated antibodies at room temperature for 30 minutes according to the manufacturer’s instruction. Fixable Viability Dye eFluor 506 (Catalog No. 65-0866-14, Invitrogen, Waltham, MA) were used for viability dye. Fluorescein isothiocyanate (FITC)-conjugated anti-mouse IL-17A monoclonal antibody (mAb) (Catalog No. 506907), APC-conjugated anti-mouse CD4 mAb (Catalog No. 100515), and BV605-conjugated anti-mouse CD8 mAb (Catalog No. 100744) were purchased from BioLegend (San Diego, CA). Samples were measured in flow cytometer, and data were analyzed with FlowJo software (FlowJo, Ashland, OR).

### 2.8 Enzyme-linked immunosorbent assay

ELISA kit (Catalog No. MBS2708370, MyBioSource, San Diego, CA) were used to determine NF-κB protein expression in mouse skin. Proteins were detected from supernatants of tissue homogenate according to the manufacturer’s instructions. Optical density (OD) value was detected with a micro-plate reader SpectraMax iD3 (Catalog No. ID3-STD, Molecular Devices, San Jose, CA). ELISA measurements were normalized to each sample’s total weight or volume as described previously (23).

### 2.9 Spatial transcriptomics

FFPE mouse skin samples that passed the RNA quality control (DV200 > 30%) were used for spatial transcriptomic library construction and sequencing. The tissues were prepared according to the Visium CytAssist Spatial Gene Expression for FFPE-Tissue Preparation Guide (CG000518, 10× Genomics, Pleasanton, CA, USA). FFPE mouse skin samples were sectioned into 10 μm, then deparaffinization, probe hybridization and probe ligation were performed. The tissue was permeabilized and the mRNA was reverse transcribed into cDNA with barcode containing slide location information on a designated area of 11 mm × 11 mm square. cDNA was then amplified and processed to obtain cDNA libraries according to the manufacturer’s protocol. To verify the size of PCR-enriched fragments, the template size distribution was checked using Agilent D1000 ScreenTape (Agilent Technologies, Santa Clara, CA). Sequencing was performed at the IGM Genomics Center at the University of California, San Diego. Libraries were sequenced using NovaSeq X Plus 10B (Illumina, San Diego, CA) with a read length of 150 – 1000 bp. Partek flow software version 11 (Partek Incorporated, Chesterfield, MO, USA) was used to process H&E images and FASTQ files and perform spatial gene expression analysis and pathway enrichment analysis. The sequencing results were guaranteed to be accurate as follows; Number of reads: IMQ-treated *S1pr2^fl/fl^*, 577,665,006; IMQ-treated *S1pr2^fl/fl^ K14-Cre*, 496,579,139; Number of spots under tissue: IMQ-treated *S1pr2^fl/fl^*, 1,213; IMQ-treated *S1pr2^fl/fl^ K14-Cre*, 975; Mean reads per spot: 903,639; 1,010,791; Median genes per spot: 1,758; 1,046.

### 2.10 Statistical analysis

In all experiments, all samples were performed in triplicates, and values were expressed as mean ± SD. Two-way ANOVA and Tukey’s multiple comparison test determined statistical significances of differences for multiple group analysis. Student *t*-test was performed to compare two groups. *P* < 0.05 was considered significant. Analyses were performed through the GraphPad Prism software version 7 (GraphPad Software, Boston, MA).

## 3 Results

### 3.1 Complete deletion of S1PR2 increased psoriasis-like inflammation in the skin

**Figure 1:**
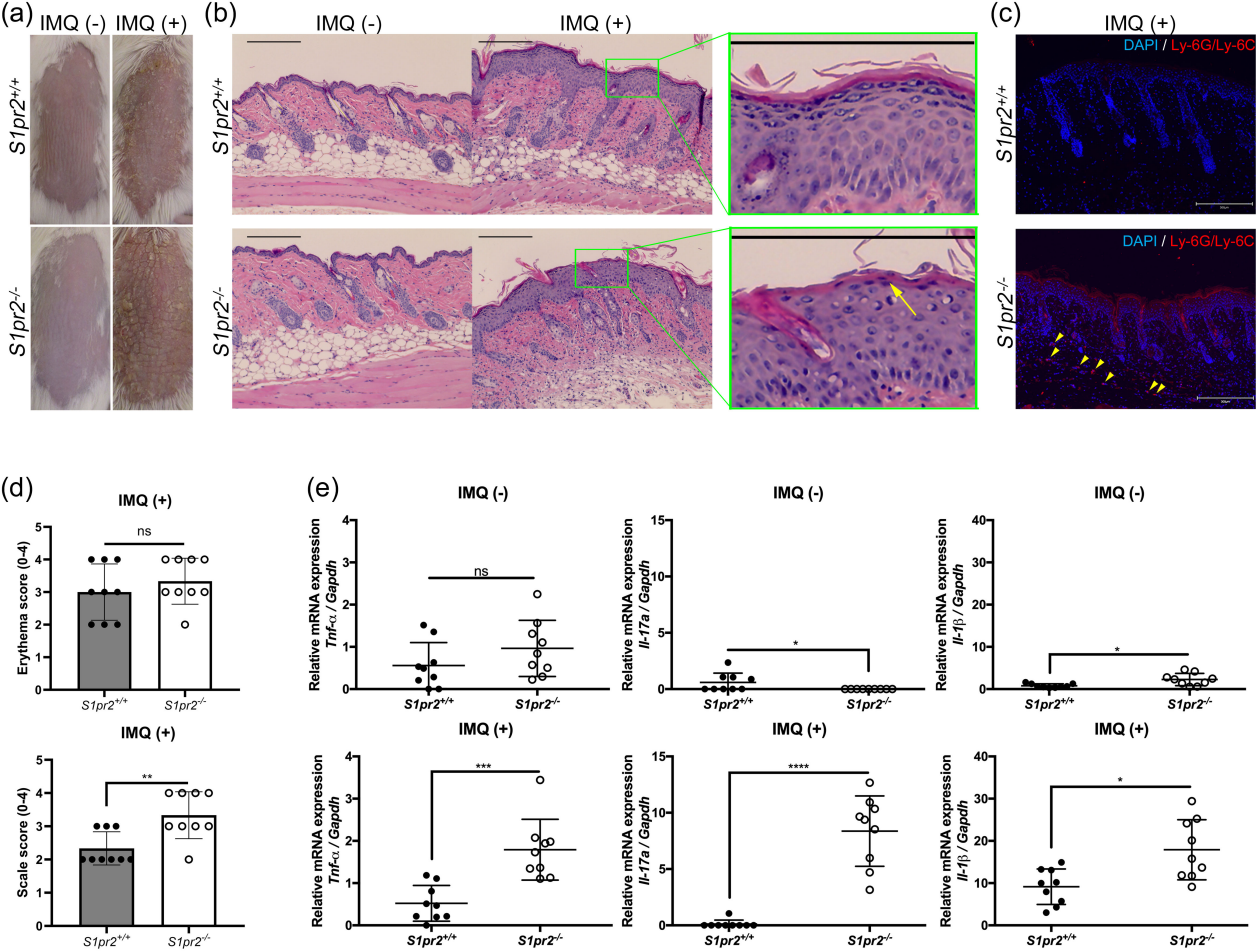
S1PR2 contributes psoriasis-like inflammation in the murine skin. **(A)** Representative clinical manifestations and **(B)** H&E staining images of dorsal skin from *S1pr2^+/+^* and *S1pr2^-/-^* mice. Arrow shows parakeratosis. **(C)** Ly-6G/Ly-6C (red) and nuclei (blue) immunofluorescence images of *S1pr2^+/+^* and *S1pr2^-/-^* mouse skin 1 week after IMQ application. Arrowhead shows Ly-6G/Ly-6C positive cells. **(D)** Severity score of erythema (upper panel) and scaling (lower panel). **(E)** mRNA expressions of *Tnf-α, Il-17a*, and *Il-1β* of Balb/c and *S1pr2^-/-^* mouse epidermis was measured with RT-qPCR and normalized to *Gapdh* mRNA expression level. Data shown are the mean ± SD and represent three independent experiments with similar results. Scale bar = 300 μm. ****p < 0.0001, ***p <0.0005, **p < 0.005, *p < 0.05. S1PR2, sphingosine 1-phosphate receptor 2; IMQ, imiquimod; ns, not significant; *Gapdh*, glyceraldehyde-3-phosphate.

To investigate whether S1PR2 has any effects on psoriasis inflammation, we induced psoriasis-like skin inflammation with 5% IMQ cream on the depilated dorsal skin of *S1pr2^-/-^* mice, which has no S1PR2 in their whole body and that of *S1pr2^+/+^* mice, *S1pr2* wild type control. After IMQ application, *S1pr2^-/-^* mice had much more scales on their dorsal skin than *S1pr2^+/+^* mice, though the severity of erythema was not different between *S1pr2^-/-^* mice and *S1pr2^+/+^* mice (**Figures 1A, D**). None developed systemic symptoms other than skin manifestations. No difference in body shape and size of the mice was observed between the *S1pr2^+/+^* mice and *S1pr2^-/-^* mice. Histological analyses with hematoxylin and eosin (H&E) staining of *S1pr2^-/-^* mice showed more parakeratosis in the stratum corneum, and more inflammatory cell infiltration in the lower dermis than that of *S1pr2^+/+^* mice after IMQ application (**Figure 1B**). In addition, immunofluorescence staining revealed that *S1pr2^-/-^* mice had more neutrophils in the lower dermis than *S1pr2^+/+^* mice after IMQ application (**Figure 1C**). We then quantified the mRNA expression levels of proinflammatory cytokines in the epidermis. We found that *S1pr2^-/-^* mice epidermis had significantly higher mRNA expressions of *Tnf-α, Il-17a*, and *Il-1β* than *S1pr2^+/+^* mice after IMQ application (**Figure 1E**). These results demonstrate the involvement of S1PR2 in psoriasis pathogenesis and suggest that deletion of S1PR2 amplifies psoriasis inflammation.

### 3.2 S1pr2 keratinocyte-specific knockout increased psoriasis-like inflammation in the skin

**Figure 2:**
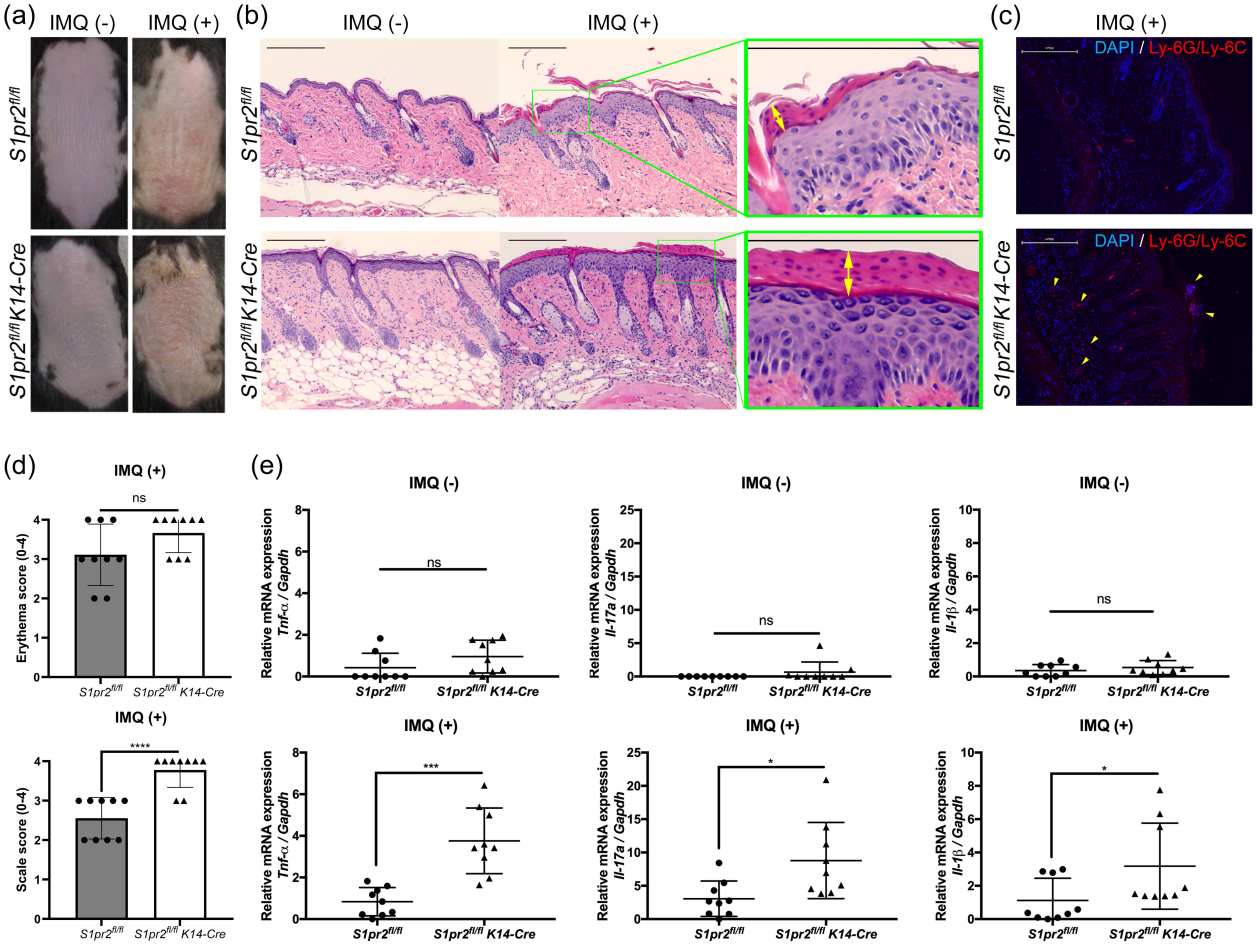
S1PR2 in keratinocytes regulates psoriasis-like inflammation in the murine skin. **(A)** Representative clinical manifestations and **(B)** H&E staining images of dorsal skin from *S1pr2^fl/fl^* and *S1pr2^fl/fl^ K14-Cre* mice. **(C)** Ly-6G/Ly-6C (red) and nuclei (blue) immunofluorescence images of *S1pr2^fl/fl^* and *S1pr2^fl/fl^ K14-Cre* mouse skin 1 week after IMQ application. Arrowhead shows Ly-6G/Ly-6C positive cells. **(D)** Severity score of erythema (upper panel) and scaling (lower panel). **(E)** mRNA expressions of *Tnf-α, Il-17a*, and *Il-1β* of *S1pr2^fl/fl^* and *S1pr2^fl/fl^ K14-Cre* mouse epidermis were measured with RT-qPCR and normalized to *Gapdh* mRNA expression level. Data shown are the mean ± SD and are representative of three independent experiments with similar results. Scale bar = 300 μm. ****p < 0.0001, ***p <0.0005, **p < 0.005, *p < 0.05. ns, not significant.

Psoriasis can be triggered by damaging stimuli such as trauma or scratching (24). Keratinocytes, which constitute the outermost layer of the skin, receive these stimuli first and directly. To assess whether the deletion of S1PR2 only in keratinocytes affects psoriasis inflammation, we induced psoriasis-like skin inflammation on the depilated dorsal skin of *S1pr2^fl/fl^ K14-Cre* mice, that lack of S1PR2 only in keratinocytes, and that of *S1pr2^fl/fl^* mice. Consistent with the results of *S1pr2^-/-^* mice, *S1pr2^fl/fl^ K14-Cre* mice showed more scales on their dorsal skin than *S1pr2^fl/fl^* mice after IMQ application, though the severity of erythema was not different between *S1pr2^fl/fl^ K14-Cre* mice and *S1pr2^fl/fl^* mice (**Figures 2A, D**). None developed systemic symptoms other than skin manifestations. No difference in body shape and size of the mice was observed between the *S1pr2^fl/fl^* mice and *S1pr2^fl/fl^ K14-Cre* mice. H&E staining of *S1pr2^fl/fl^ K14-Cre* mice presented more parakeratosis in the stratum corneum and more inflammatory cell infiltration in the lower dermis than that of *S1pr2^fl/fl^* mice (**Figure 2B**). Immunofluorescence staining analysis showed that *S1pr2^fl/fl^ K14-Cre* mice had more neutrophils in the lower dermis than *S1pr2^fl/fl^* mice (**Figure 2C**). We investigated proinflammatory cytokine profiles and found that *S1pr2^fl/fl^ K14-Cre* mice epidermis had significantly higher mRNA expressions of TNF-α, Il-17A, and IL-1β than that of *S1pr2^fl/fl^* mice after IMQ application (**Figure 2E**).

Several studies on mice revealed that the S1PR modulator fingolimod (FTY720) binds S1PR1, 3, 4, and 5, and selective S1PR1 modulator Syl930 ameliorates psoriasis inflammation (12, 25). We, therefore, investigated whether all other S1PRs could regulate inflammation in psoriasis exacerbated by S1PR2 deficiency in keratinocytes. We treated IMQ-induced psoriasis skin of *S1pr2^fl/fl^* and *S1pr2^fl/fl^ K14-Cre* mice with a topical application of FTY720 and compared cytokine profiles with control and IMQ-only groups (**Supplementary Figure S1A**). With FTY720 and IMQ, *S1pr2^fl/fl^ K14-Cre* mice still had more scaly erythema than *S1pr2^fl/fl^* mice (**Supplementary Figures S1B, C**), and *S1pr2^fl/fl^ K14-Cre* mice epidermis had significantly higher mRNA expressions of TNF-α and IL-1β than that of *S1pr2^fl/fl^* mice. These results suggest that S1PR2 in keratinocytes plays a crucial role in regulating inflammation in psoriasis and that S1PR2 is more effective in controlling psoriasis than other S1PRs.

### 3.3 S1pr2 keratinocyte-specific knockout increased Th17 cells in psoriasis-like skin-draining lymph nodes

**Figure 3:**
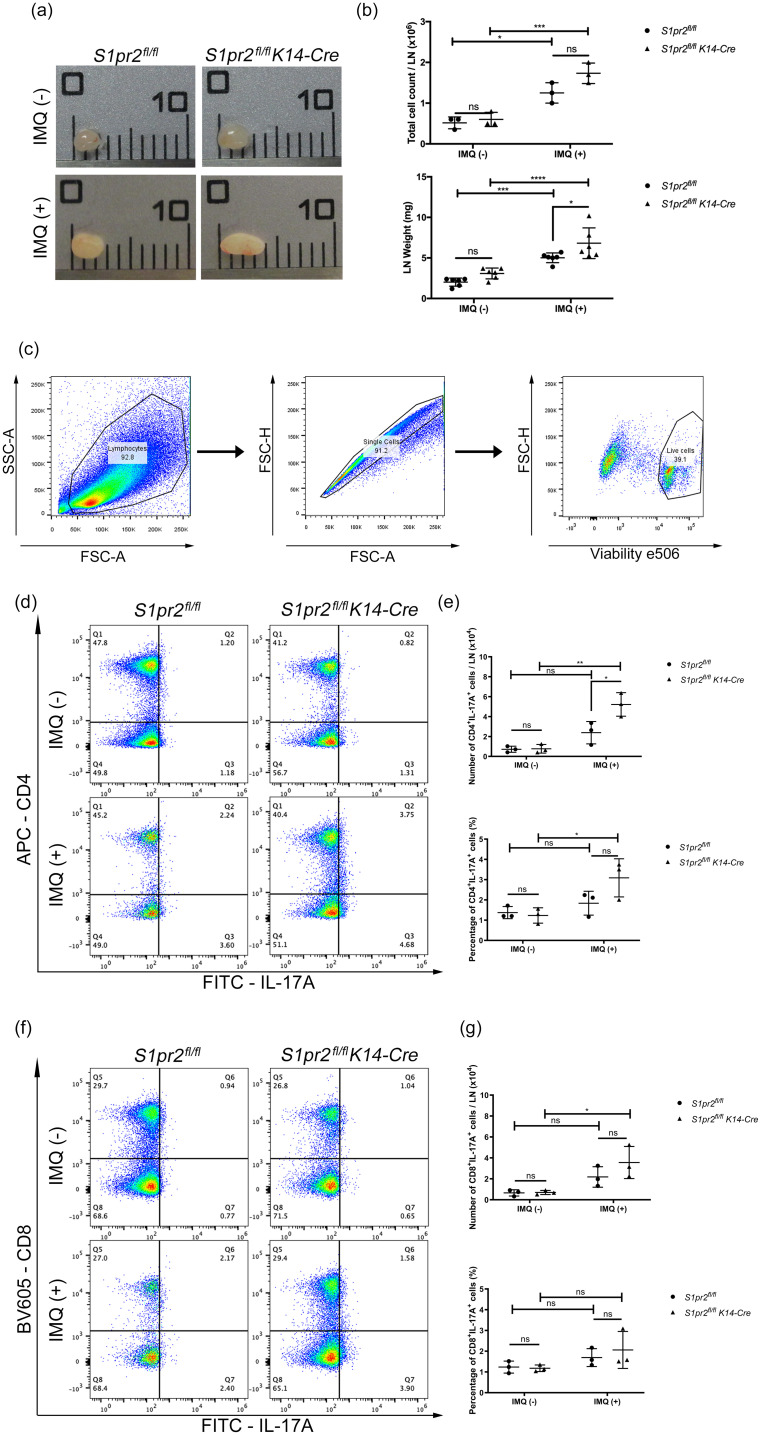
S1PR2 deletion in keratinocytes increases Th17 cell population in skin-draining lymph nodes. **(A)** Representative images, **(B)** total cell count and weight of skin-draining lymph nodes of IMQ-treated *S1pr2^fl/fl^* and *S1pr2^fl/fl^ K14-Cre* mice. In each group, a total of six axillary LNs were obtained from the right and left armpit from three mice. Caliper grid line distance is 1 mm. **(C, D, F)** Flow cytometry analysis of skin-draining LN cells of IMQ-treated *S1pr2^fl/fl^* and *S1pr2^fl/fl^ K14-Cre* mice. The number in each quadrant represents the percentage of the cells. **(E, G)** Bar graph shows the population of CD4^+^IL-17A^+^ cells and CD8^+^IL-17A^+^ cells among live cells as measured by flow cytometry analysis **(C, D, F)**. LN, lymph node.

IL-17A plays a critically important role in psoriasis inflammation, and Th17 cells are thought to be the main source of IL-17A in psoriasis (26). To investigate whether S1PR2 deletion in keratinocytes affects Th17 cell increase in psoriasis-like skin, we examined the distribution of CD4^+^ IL-17A^+^ and CD8^+^ IL-17A^+^ T cells in the skin-draining LNs from *S1pr2^fl/fl^ K14-Cre* and *S1pr2^fl/fl^* mice after IMQ application. In terms of size and weight of LNs, *S1pr2^fl/fl^ K14-Cre* mice had larger and heavier LNs than *S1pr2^fl/fl^* mice (**Figures 3A, B**). In addition, we observed a larger number of CD4^+^IL-17^+^ T cells in the skin-draining LNs of *S1pr2^fl/fl^ K14-Cre* mice than that of *S1pr2^fl/fl^* mice after IMQ administration, while no change was observed in the population of CD8^+^IL-17^+^ T cells between *S1pr2^fl/fl^ K14-Cre* and *S1pr2^fl/fl^* mice (**Figures 3C–G**). We also examined the distribution of *Cd4* ^+^ *Il-17a* ^+^ and *Cd8a* ^+^ *Il-17a* ^+^ areas in the skin by spatial sequencing to investigate the population of Th17 cells and Tc17 cells in the skin. Contrary to the LN results, a lower percentage of Cd4+Il-17a+ areas and a lower percentage of *Cd8a* ^+^ *Il-17a* ^+^ areas were observed in the IMQ-treated *S1pr2^fl/fl^ K14-Cre* mouse skin, compared to *S1pr2^fl/fl^* mouse skin (**Supplementary Figures S2A, B**). Next, we examined Cd2+Cd3+Il-17a+ areas to investigate the distribution of γδ T cells and observed a smaller number in the IMQ-treated *S1pr2^fl/fl^ K14-Cre* mouse skin (**Supplementary Figure S2C**). It has been reported that IL-17A production by CD8+ T cells is upregulated in psoriatic skin (27), and the results of this study suggest that S1PR2 deficiency increases IL-17A production from CD8+ cells in the psoriatic skin region. These results suggest that deleting S1pr2 in mouse keratinocytes affects skin-resident cells and the recruitment of IL-17-producing cells during psoriasis-like inflammation.

### 3.4 S1pr2 deletion in keratinocytes is not involved in alterations of skin barrier function in psoriasis-like inflammation

The potential involvement of skin barrier dysfunction in the pathogenesis of psoriasis has been previously discussed (28). Some reports showed an increase of tight junction proteins and filaggrin in psoriasis lesions (29, 30), while others showed a decrease (31, 32). Thus, a consensus has yet to be reached. Since our previous studies have shown that S1PR2 is essential for maintaining and recovering epidermal skin barrier function (13, 16), we investigated whether the skin barrier is altered in psoriasis-like inflammation lacking epidermal S1PR2. RT- qPCR analysis showed no specific trend in mRNA expression levels of the skin barrier proteins between the control and the IMQ-treated group in either *S1pr2^fl/fl^* or *S1pr2^fl/fl^ K14-Cre* mice (**Supplementary Figure S3**). Our results suggest that impairment of the skin barrier function is not involved in the pathogenesis of psoriasis.

### 3.5 S1pr2 deletion and S1pr2 keratinocyte-specific knockout lead to a much greater increase in the MyD88/NF-κB pathway compared to the wild type

**Figure 4:**
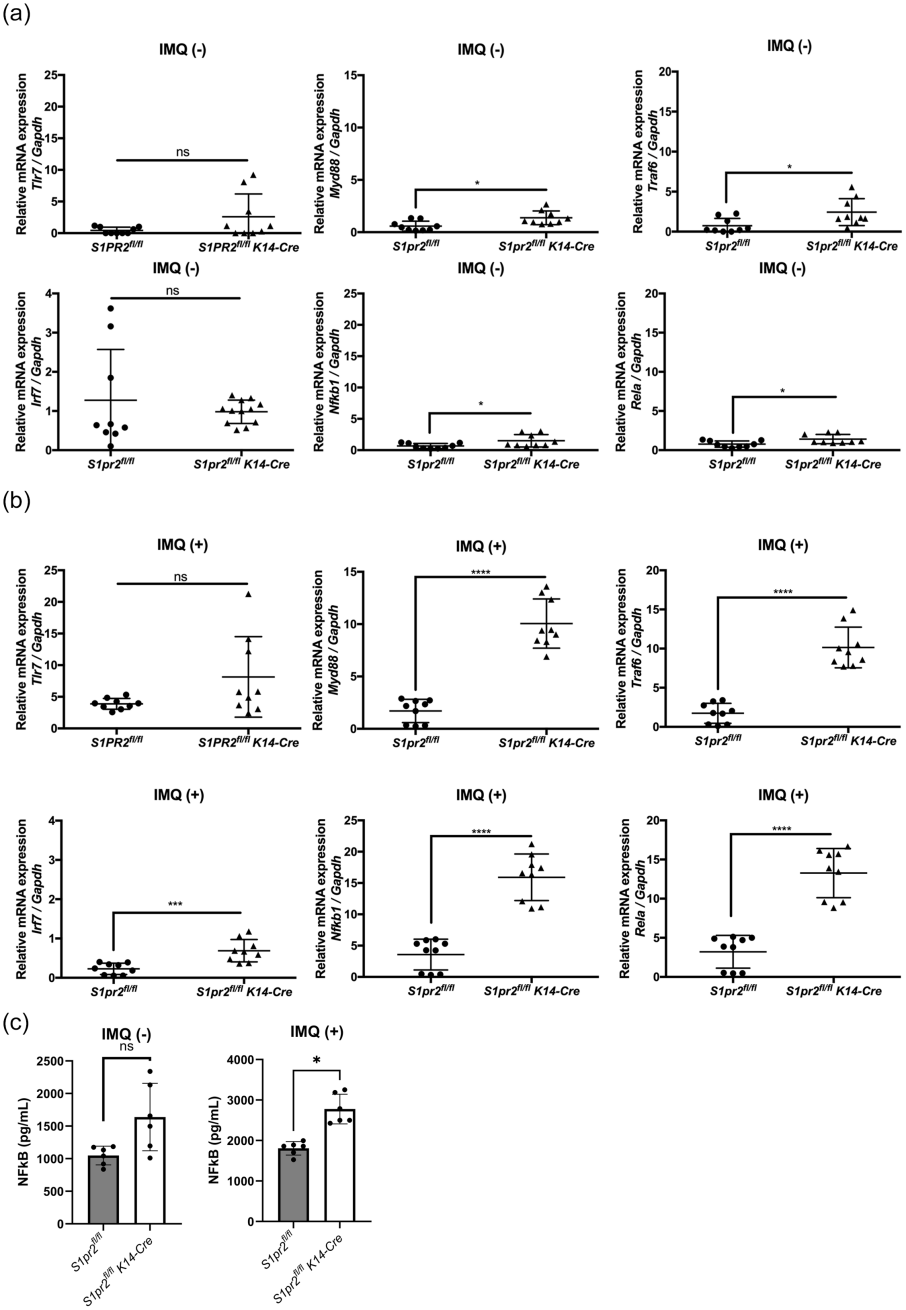
S1PR2 deletion increases MyD88/NF-κB pathway molecules but not affects TLR7 expression. mRNA expressions of *Tlr7, Myd88*, *Traf6, Irf7, Nfkb1*, and *Rela* of **(A)** Balb/c and *S1pr2^-/-^* mouse epidermis, and **(B)** *S1pr2^fl/fl^* and *S1pr2^fl/fl^ K14-Cre* mouse epidermis were measured with RT-qPCR and normalized to *Gapdh* mRNA expression level. **(C)** The protein level of NF-κB was measured by enzyme-linked immunosorbent assay. Data shown are the mean ± SD (n = 3) and are representative of three independent experiments with similar results. ****p < 0.0001, ***p <0.0005, **p < 0.005, *p < 0.05. ns, not significant.

In a mouse model of psoriasis, IMQ acts as a TLR7 ligand to induce the expression of cytokines required for activation of the Th17 pathway and inflammatory factors necessary for psoriasis development (33). To explore how S1PR2 affects IMQ-induced psoriasis-like inflammation, we examined mRNA expressions of the TLR7 signaling pathway in the epidermis of S1PR2-deficient mice. In the epidermis of IMQ-treated *S1pr2^-/-^* mice, mRNA levels of *Myd88, Irf7, Nfkb1*, and *Rela* were higher than in wild-type mice, but no significant difference was observed in the mRNA level of *Tlr7* (**Figure 4A**). Similar to that, the epidermis of IMQ-treated *S1pr2 ^fl/fl^ K14-Cre* mice had higher mRNA levels of *Myd88, Traf6, Irf7, Nfkb1*, and *Rela* compared to *S1pr2 ^fl/fl^* mice (**Figure 4B**). In addition, the epidermis of IMQ-treated *S1pr2 ^fl/fl^ K14-Cre* mice had higher protein levels of NF-κB (**Figure 4C**). This result shows that the deletion of S1PR2 causes an excessive increase in the MyD88/NF-κB pathway, not solely due to TLR7 stimulation.

### 3.6 Spatial transcriptomics revealed that S1pr2 deletion in keratinocytes increases the MyD88 / NF-κB pathway and psoriasis-related cytokine expression in the epidermis

**Figure 5:**
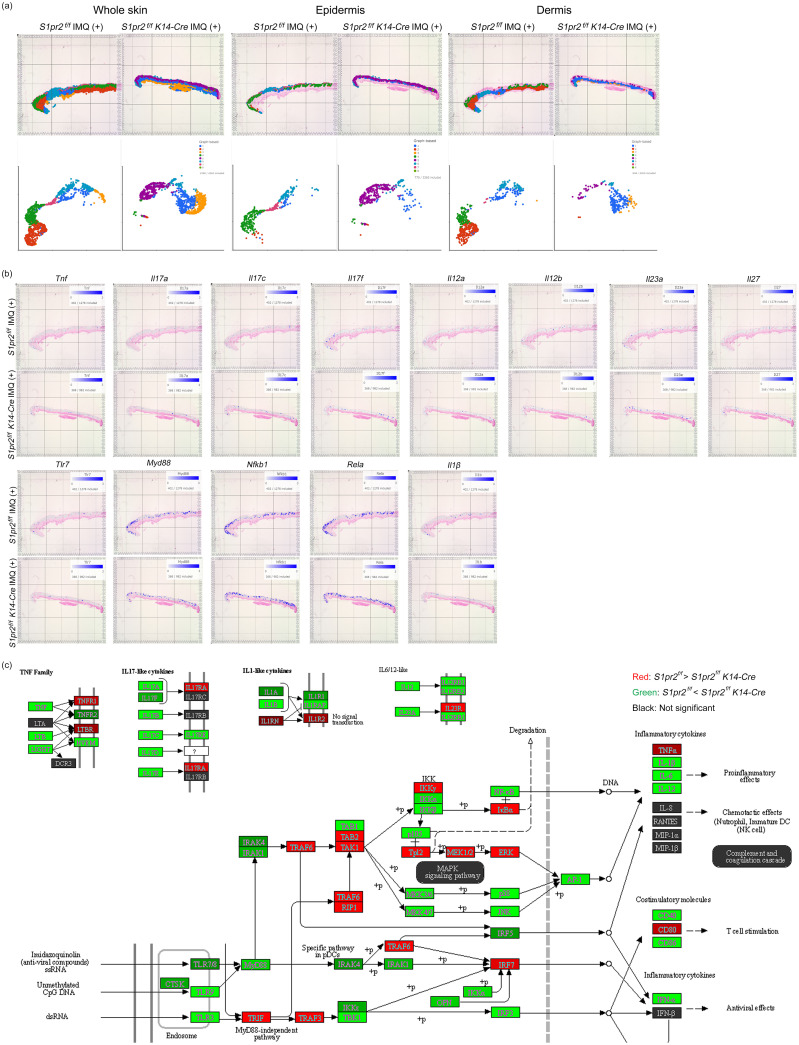
Spatial transcriptomics reveals that S1PR2 deletion in keratinocytes results in psoriasis-like inflammation in the epidermis. **(A)** Graph-based clustering (upper lane) and UMAPs (lower lane) of *S1pr2 ^fl/fl^* and *S1pr2 ^fl/fl^ K14-Cre* mouse epidermis after IMQ administration. **(B)** Spatial gene expression analysis of *Tnf, Il-17a, Il-17c, Il-17f, Il-12a, Il-12b, Il-23a, Il-27, Tlr7, Myd88, Nfkb1*, *Rela, and Il-1β* in *S1pr2 ^fl/fl^* and *S1pr2 ^fl/fl^ K14-Cre* mouse epidermis after IMQ administration. **(C)** Representatives of pathway enrichment analysis of the mRNA levels by spatial transcriptomics of *S1pr2 ^fl/fl^* and *S1pr2 ^fl/fl^ K14-Cre* mouse epidermis after IMQ administration. Genes upregulated and downregulated in *S1pr2 ^fl/fl^ K14-Cre* mouse epidermis are highlighted in green and red, respectively. Fold change > 2 and adjusted P value < 0.05.

To confirm histology and qPCR analysis, we performed spatial transcriptomics of whole skin tissue from IMQ-treated *S1pr2 ^fl/fl^* and *S1pr2 ^fl/fl^ K14-Cre* mouse. Graph-based clustering analysis and Uniform Manifold Approximation and Projection for Dimension Reduction (UMAP) showed that epidermis and dermis were classified in separate clusters for IMQ-treated *S1pr2 ^fl/fl^* and *S1pr2 ^fl/fl^ K14-Cre* mouse, although subcutaneous tissue was classified as a same cluster in both IMQ-treated *S1pr2 ^fl/fl^* and *S1pr2 ^fl/fl^ K14-Cre* mouse (**Figure 5A**). In addition, the spatial gene expression analysis showed an upregulation of *Tnf, Il-17a*, *Il-17c*, *Il-17f*, and *Il-1β* in the epidermis of IMQ-treated *S1pr2 ^fl/fl^ K14-Cre* mouse, consistent with the findings from the pathway enrichment analysis (**Figures 5B, C**). In addition, gene expression levels of Il-12 cytokine families such as *Il-12a, Il-12b, Il-23a*, and *Il-27* were upregulated in the epidermis of IMQ-treated *S1pr2 ^fl/fl^ K14-Cre* mouse (**Figures 5B, C**). These data support the fact that the IL-23/Th17 axis is recognized as a key link in the immunopathogenesis of psoriasis, and S1P has been reported to affect IL-23-mediated signaling (34). Moreover, the spatial gene expression analysis and pathway enrichment analysis revealed that the MyD88/NF-κB pathway is upregulated in IMQ-treated *S1pr2 ^fl/fl^ K14-Cre* mouse epidermis, while TLR domain-containing adapter inducing IFN-β (TRIF) and TRAF3, a MyD88-independent signaling pathway, is downregulated (**Figure 5C**, lower panel). These results support the histology and qPCR findings that S1PR2 deletion in keratinocytes induces MyD88/NF-κB pathway increase in the epidermis, resulting in proinflammatory cytokine increase after IMQ stimulation. Since angiogenesis is an exacerbating factor of psoriatic inflammation (35), we also investigated the gene expression levels of vascular endothelial growth factor (VEGF), hypoxia-inducible factor (HIF), the murine IL-8 homolog CXCL1, and angiopoietin that are considered to be the main players in angiogenesis (**Supplementary Figure S4**). The spatial gene expression analysis and pathway enrichment analysis showed that S1PR2 deletion did not alter these expressions or even reduce them. This is consistent with the fact that S1PR2 does not change the severity of erythema, suggesting that epidermal S1PR2 does not affect angiogenesis.

### 3.7 S1PR2 regulates psoriasis-like inflammation in human keratinocytes

#### 3.7.1 Human epidermal keratinocytes express TLR7

**Figure 6:**
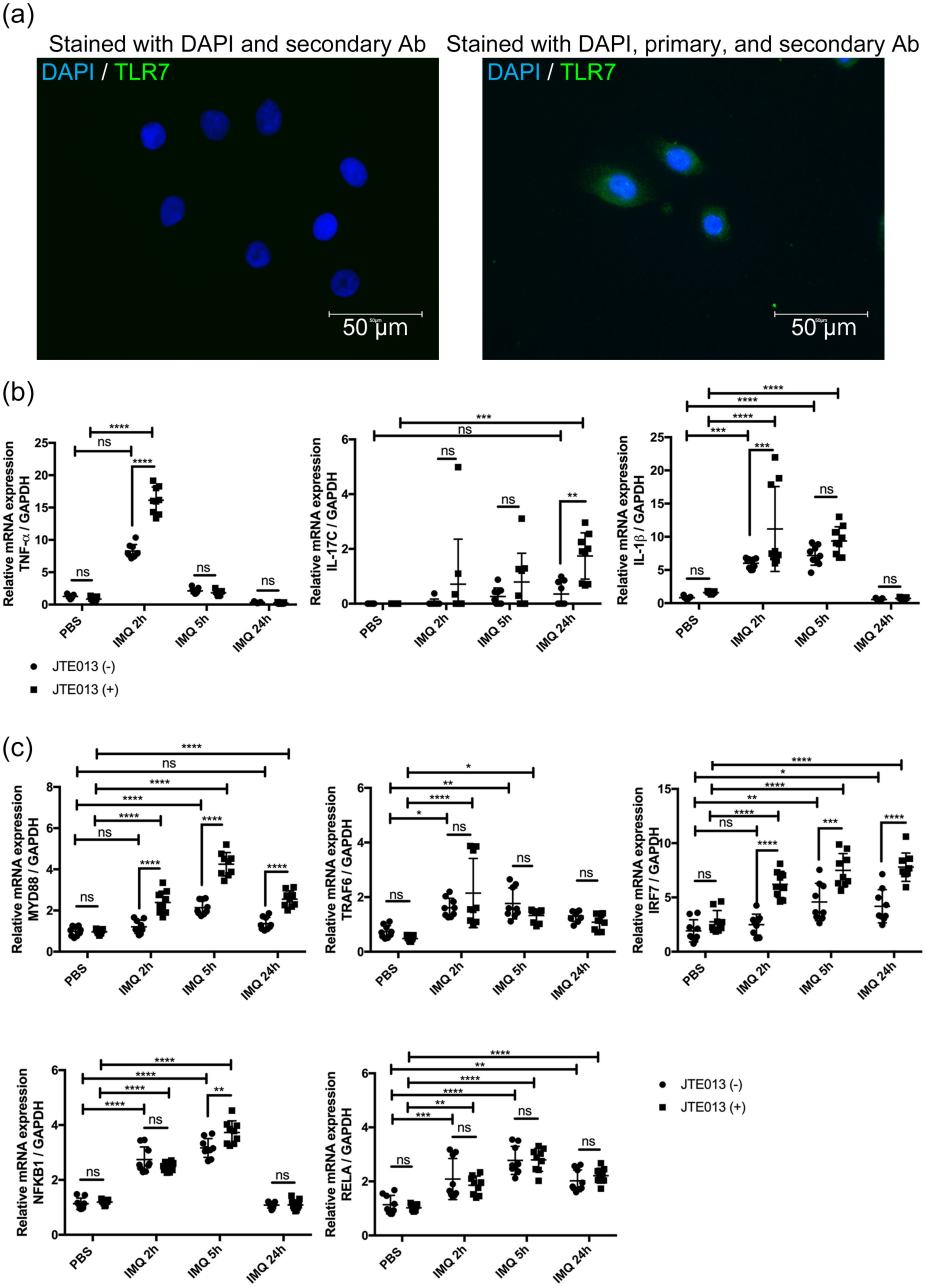
S1PR2 blockade in NHEK increases psoriasis-related inflammation. **(A)** Representative immunofluorescence images of TLR7 (green) and nuclei (blue) of NHEKs. Left panel shows NHEKs stained with DAPI and secondary antibody. Right panel shows NHEKs stained with DAPI, primary, and secondary antibody **(B)** mRNA expressions of TNF-α, Il-17C, IL-1β, and **(C)** mRNA expressions of MYD88, TRAF6, IRF7, NFKB1, and RELA of NHEKs treated with PBS for 24 hours or with IMQ for 2, 5, and 24 hours. Before IMQ treatment, S1PR2 was inhibited by incubating NHEKs with 10 μM JTE013 for 2 hours. Each gene expression was measured with RT-qPCR and normalized to GAPDH mRNA expression level. Data shown are the mean ± SD (n = 3) and are representative of three independent experiments with similar results. ****p < 0.0001, ***p <0.0005, **p < 0.005. ns, not significant.

It was previously stated that NHEKs express TLRs 1, 2, 3, 4, 5, 6, and 9 but not TLR7 and TLR8 (36–38). However, a recent study found that human keratinocytes express TLR7 after calcium-induced differentiation (21). Since the expression of TLR7 is an essential factor in whether the IMQ is an appropriate reagent to induce psoriasis-like inflammation in NHEKs, we performed immunofluorescence staining on NHEKs to confirm that they express TLR7. According to the immunofluorescence staining analysis, we found that NHEKs expressed TLR7 and concluded that NHEKs could be stimulated with IMQ to induce a psoriatic condition similar to that of keratinocytes in a psoriasis mouse model (**Figure 6A**).

#### 3.7.2 S1PR2-blocked NHEKs have higher proinflammatory cytokines after IMQ stimulation

We next blocked S1PR2 on NHEKs using JTE013 and stimulated them with IMQ to investigate whether S1PR2 in human keratinocytes affects in the same way as in the murine model in psoriasis inflammation. Consistent with mouse model results, S1PR2 blockade of NHEKs significantly increased mRNA expressions of TNF-α at 2 hours, IL-1β at 5 hours, and IL-17C at 24 hours after IMQ administration (**Figure 6B**). Furthermore, in S1PR2 blocked NHEKs, mRNA expressions of MyD88 and IRF7 were observed at any time after IMQ administration, and that of NFKB1 was observed 5 hours after IMQ administration (**Figure 6C**). Altogether our *in vivo* and *in vitro* data indicate a critical role for keratinocyte S1PR2 in psoriasis-like inflammation through regulating MyD88/NF-κB pathway.

## 4 Discussion

S1P is one of the sphingolipids produced via the degradation of ceramide and the phosphorylation of sphingosine acts as a signaling molecule by binding receptors S1PRs (S1PR1-5) (7). Each S1PR is known to have a different function on cellular responses, such as cell proliferation, differentiation, apoptosis, and immune responses (39). Among the five S1PRs, S1PR1 has been primarily studied for its association with psoriasis (35, 40). In addition, fingolimod, an S1PR1 functional antagonist that binds to S1PR3-5, has been reported to inhibit the migration of T cells from the LN to the skin and alleviate IMQ-induced psoriasis (12). However, the role of S1PR2 in psoriasis has yet to be investigated. Our study demonstrated that S1PR2 deletion increases psoriasis inflammation. Namely, our data indicated that S1PR2 acts as an immunosuppressor in psoriasis pathogenesis.

Since the discovery that T cells play a major role in the pathogenesis of psoriasis, research on the pathogenesis and treatment of psoriasis has primarily targeted immune cells (41–45). However, the importance of crosstalk between keratinocytes and immune cells in psoriasis has received increasing attention recently (4, 46). In psoriasis lesions, external stimulation activates keratinocytes to produce proinflammatory cytokines such as TNF-α and IL-1β, stimulating dendritic cells to induce differentiation of helper T cells (47–49). One of the helper T cells subsets the Th17 cell, produces IL-17A, and stimulates the keratinocytes to produce proinflammatory cytokines such as IL-1β, IL-17C, and IL-8, which induces the recruitment of neutrophils (2, 50–52). IL-1β secreted from keratinocytes acts on keratinocytes to promote cytokine production via the MyD88/NF-κB pathway (52, 53). This positive feedback forms a loop to maintain the inflammatory phase of psoriasis. Our data demonstrated that S1PR2 in keratinocytes interacts with the MyD88/NF-κB pathway and regulates this feedback loop.

In studies other than the skin and keratinocytes, S1PR2 has been reported to have pro-inflammatory functions. For example, vascular endothelial cell inflammation is suppressed in S1PR2-deficient mice (54), and osteoclast formation is inhibited in S1PR2-deficient mice (55). On the other hand, it has been reported that S1PR2 acts as a suppressive mediator in tumors; S1PR2 signaling confers tumor suppressive activity in diffuse large B-cell lymphoma (56), and lower S1PR2 values are associated with a worse prognosis in cervical squamous cell carcinoma (57).

Several studies have reported that S1PR2 ameliorates atopic dermatitis in skin diseases by promoting the Th2 cell-attracting capacity of dendritic cells (58, 59). However, another study has shown that S1PR2 deficiency increases skin inflammation by causing hyperpermeability and exacerbating mechanical stress damage and bacterial penetration (13). Our data showed that the absence of S1PR2 in keratinocytes worsened skin inflammation in psoriasis. This suggests that S1PR2 is anti-inflammatory in keratinocytes, in contrast to its effects on other cell types.

In addition, although several studies reported that FTY720, a functional antagonist of S1PR1, 3, 4, and 5, ameliorated psoriasis, the study in mice found that topical FTY720 does not alter psoriasis inflammation (19). This was hypothesized to be due to S1PR2’s function restraining hyperplasia in the skin (14, 60). Our data showed that the exacerbation of psoriasis caused by S1PR2 deficiency was not ameliorated by the administration of FTY720. This supports the notion that S1PR2 has anti-inflammatory properties in keratinocytes, and psoriasis worsens when absent. Furthermore, the study suggests that the absence of S1PR2 signaling cannot be compensated for by modulating other S1PRs. Since our previous study revealed that S1PR2 has a crucial role in skin barrier maintenance (13), we investigated the relationship between skin barrier function and psoriasis inflammation, which has been discussed in recent years (28). Of note, the association between skin barrier function and psoriasis inflammation is not consistent, with some clinical studies showing increased expression of tight junction proteins in the psoriasis skin (29, 61), while some clinical studies have shown decreased tight junction proteins and filaggrin expression in psoriasis (31, 32). In addition, a study that analyzed filaggrin and tight junction proteins in human psoriasis, ex vivo models, and *in vitro* models found that tight junction changes were observed in the early stage of psoriasis but not in developed psoriasis (30). This study shows that S1PR2 in keratinocytes inhibits psoriasis inflammation due to the MyD88/NF-κB pathway but not due to the skin barrier impairment. Since our data did not show any correlation between skin barrier and psoriasis inflammation, we support the idea that skin barrier changes are not responsible for psoriasis cytokine production.

MyD88 is an adaptor protein that signals downstream of TLRs and IL-1 receptor family members and activates the transcription factor NF-κB (62, 63). Thus, MyD88 is a central molecule that regulates immune responses in many cell types. However, MyD88 does not elicit the same response in different cell types. MyD88-mediated TLR signaling induces the secretion of Th1 and Th2 cytokines in mast cells (64). On the other hand, MyD88 in keratinocytes regulates Th1 and Th17 but not Th2 responses in allergic reactions (65). In addition, developing atopic and contact dermatitis requires MyD88 signaling in dendritic cells but not in keratinocytes (65–67). In our previous study, S1PR2 deletion in keratinocytes increased mRNA expressions of *Il-1β* and *Cxcl1* of the contact dermatitis mouse model but did not change other cytokines and clinical manifestations (16). The difference between psoriasis and contact dermatitis is due to the specific regulation of the MyD88/NF-κB pathway by S1PR2 in keratinocytes. Thus, the mechanism we found applies only to psoriasis inflammation.

A possible mechanism for S1PR2 to suppress the MyD88/NF-κB pathway is its balance with the MyD88-independent TLR signaling pathway. Our spatial transcriptomic analysis revealed that in the presence of S1PR2, MyD88-independent TLR signaling pathways such as TRIF and TRAF3 are activated when stimulated with IMQ, although the MyD88/NF-κB pathway is downregulated. However, further studies are required to determine the cause of this imbalanced signaling pathway.

In addition, this study’s limitation is that it did not focus on immune cells such as DCs or macrophages and analyzed specifically the Th17 cell population. Since DCs and macrophages are also key players in the immune response closely associated with psoriasis pathogenesis, these cells should be investigated in the future. Moreover, it is necessary to study the spleen, a tissue where immune cells are assembled, in order to further investigate the effects on systemic inflammation.

Our study is groundbreaking because it is the first to show that a signal generated by a lipid in keratinocytes can directly generate psoriatic changes in the skin. We followed the effects of the S1P signal generated by the keratinocytes and how it reverberates throughout the skin.

In summary, we demonstrated that S1PR2 is involved in regulating psoriasis inflammation and, in particular, that S1PR2 in keratinocytes plays an essential role in this regulation. This study emphasizes the significance of keratinocytes in psoriasis research and suggests targeting S1PR2 as a novel treatment approach.

## Data Availability

The datasets generated and analyzed for this study can be found at https://www.ncbi.nlm.nih.gov/bioproject/PRJNA1161425, hosted by National Center for Biotechnology Information (NCBI), accession number PRJNA1161425; GEO: GSE277246.
