# Supplementary material for: Sphingosine 1-phosphate receptor 2 in keratinocytes plays a key role in reducing inflammation in psoriasis
Source: Front Immunol. 2024 Sep 26;15:1469829. doi: 10.3389/fimmu.2024.1469829 (PMC11464331; doi:10.3389/fimmu.2024.1469829)
Supplement: Supplementary file 1 [file DataSheet1.pdf]

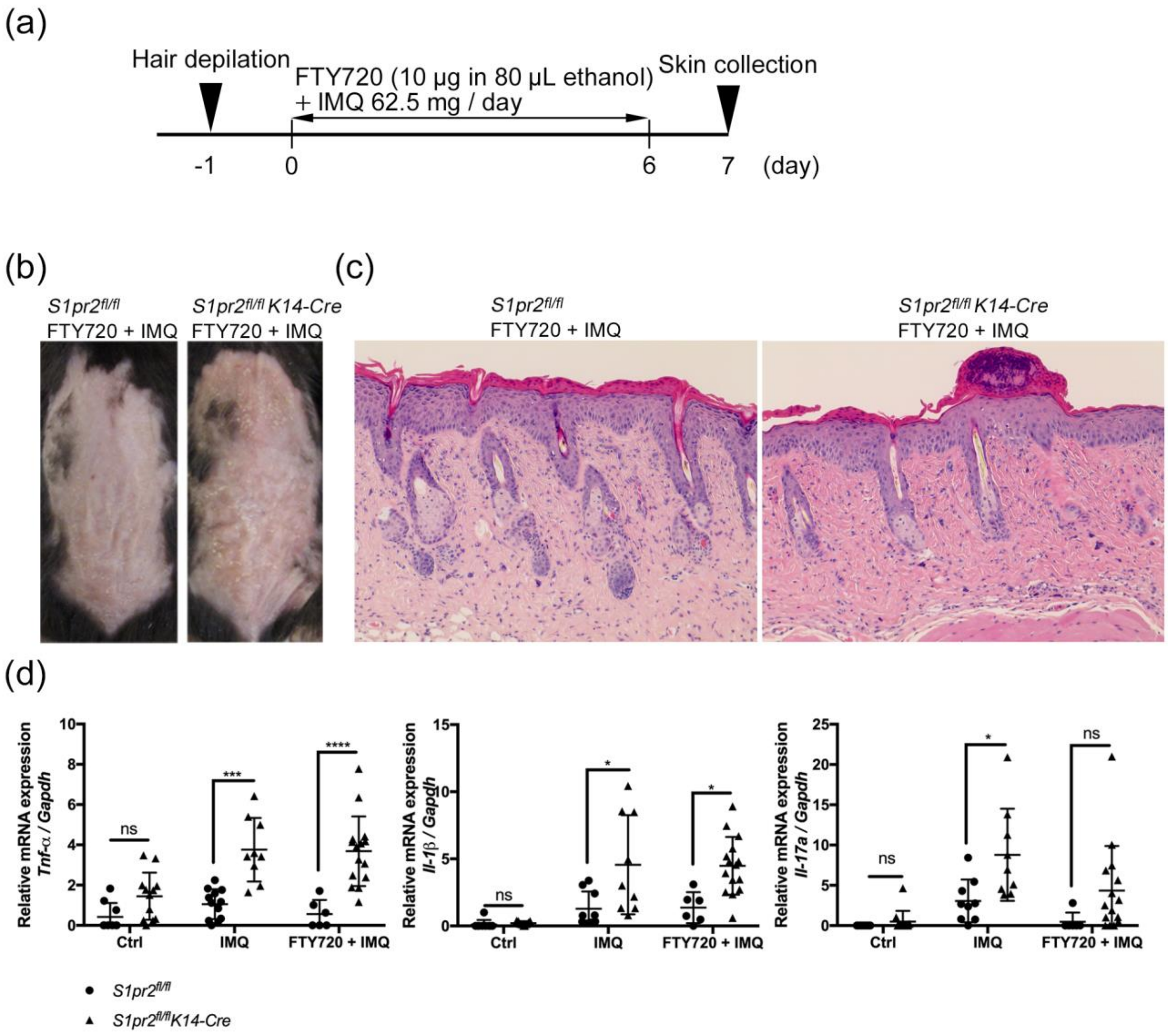

Supplementary Figure S1. Fingolimod (FTY720) does not alter psoriasis inflammation exacerbated by S1PR2 deletion in keratinocytes

(a) The experimental protocol for the FTY720 administration and IMQ application of the psoriasis mouse model. (b) Representatives of *S1pr2*$^{fl/fl}$ and *S1pr2*$^{fl/fl}$ *K14-Cre* mice treated with FTY720 and IMQ. (c) H&E staining images of *S1pr2*$^{fl/fl}$ and *S1pr2*$^{fl/fl}$ *K14-Cre* epidermis. (d) mRNA expressions of *Tnf-α, Il-17a*, and *Il-1β* of *S1pr2*$^{fl/fl}$ and *S1pr2*$^{fl/fl}$ *K14-Cre* mouse epidermis were measured with RT-qPCR and normalized to *Gapdh* mRNA expression level. ****$p < 0.0001$, ***$p < 0.0005$, *$p < 0.05$. FTY720, fingolimod; IMQ, imiquimod; ns, not significant.

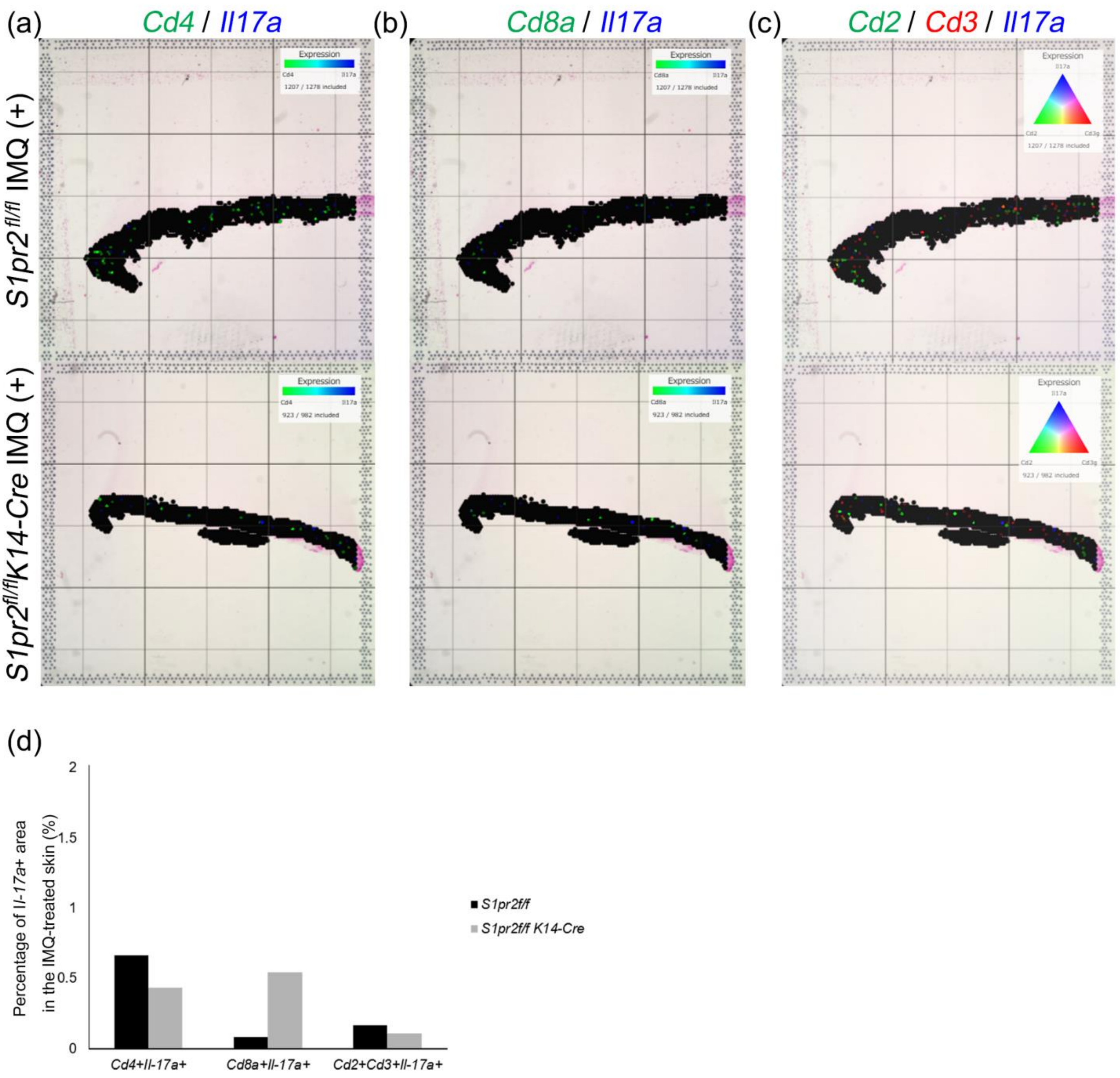

Supplementary Figure S2. ***Cd4***$^{+}$***Il-17a***$^{+}$ **(Th17),** ***Cd8a***$^{+}$***Il-17a***$^{+}$ **(Tc17), and** ***Cd2***$^{+}$***Cd3***$^{+}$***Il-17a***$^{+}$ **(γδT) percentage in the skin after IMQ application.**

Gene expression analysis by spatial sequencing shows (a) *Cd4*$^{+}$*Il-17a*$^{+}$ (bright green), (b) *Cd8a*$^{+}$*Il-17a*$^{+}$ (bright green), and (c) *Cd2* $^{+}$*Cd3* $^{+}$ *Il-17a*$^{+}$ (white) areas in the skin of *S1pr2*$^{fl/fl}$ and *S1pr2*$^{fl/fl}$ *K14-Cre* mouse after IMQ application. (d) Bar graph shows percentages of *Cd4*$^{+}$*Il-17a*$^{+}$, *Cd8a*$^{+}$*Il-17a*$^{+}$, and *Cd2* $^{+}$*Cd3* $^{+}$ *Il-17a*$^{+}$ areas in the skin of *S1pr2*$^{fl/fl}$ and *S1pr2*$^{fl/fl}$ *K14-Cre* mouse after IMQ application as measured by gene expression analysis.

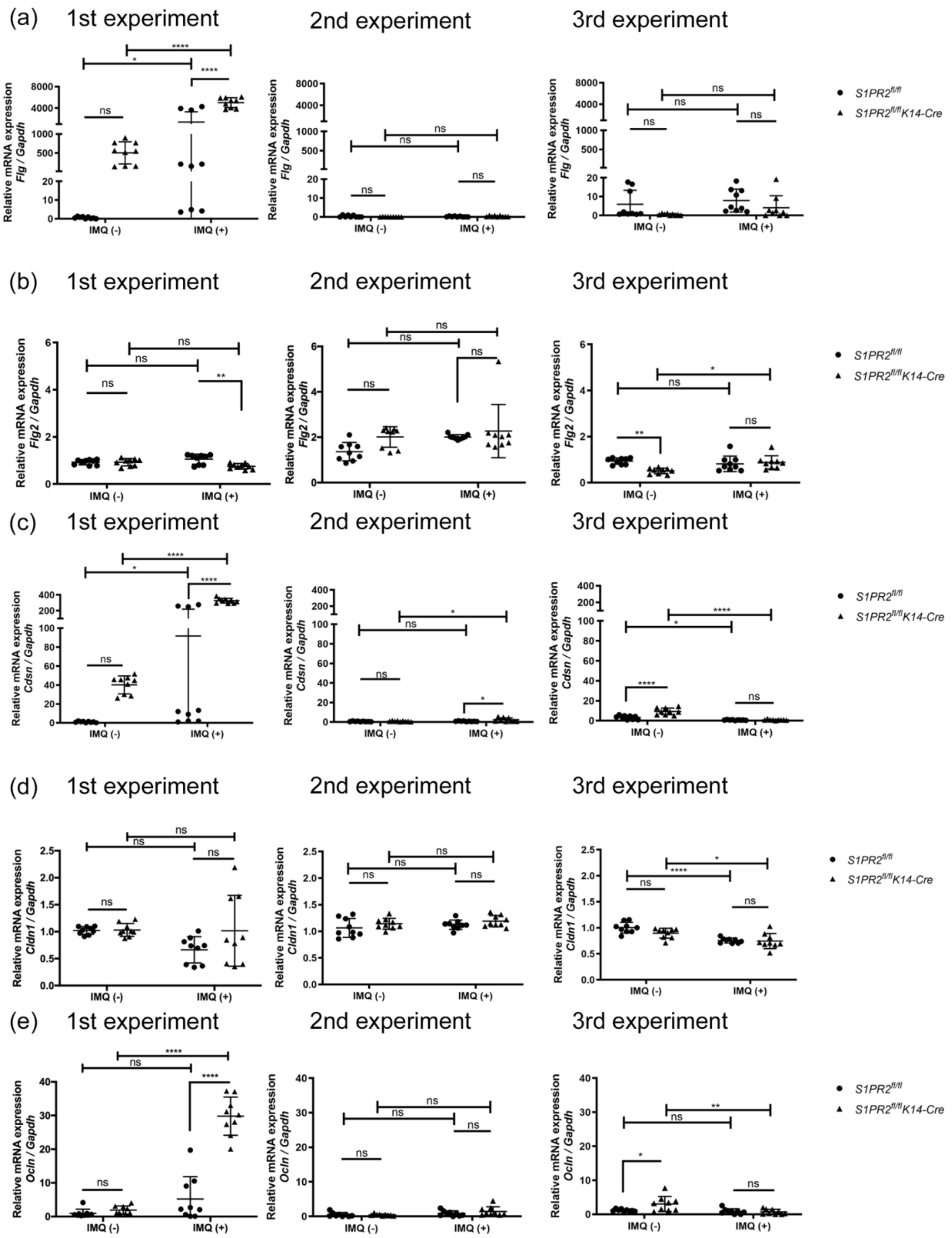

Supplementary Figure S3. mRNA expressions of epidermal barrier proteins

mRNA expressions of (a) *Flg*, (b) *Flg2*, (c) *Cdsn*, (d) *Cldn1*, *and* (e) *Ocln* of *S1pr2*$^{fl/fl}$ and *S1pr2*$^{fl/fl}$ *K14-Cre* mouse epidermis were measured with RT-qPCR and normalized to *Gapdh* mRNA expression level. Data shown are the mean ± SD (n = 3) of three independent experiments. ****$p < 0.0001$, ***$p < 0.0005$, **$p < 0.005$, *$p < 0.05$. *Flg*, filaggrin; *Cdsn*, corneodesmosine; *Cldn*, claudin; *Ocln*, occludin; ns, not significant.

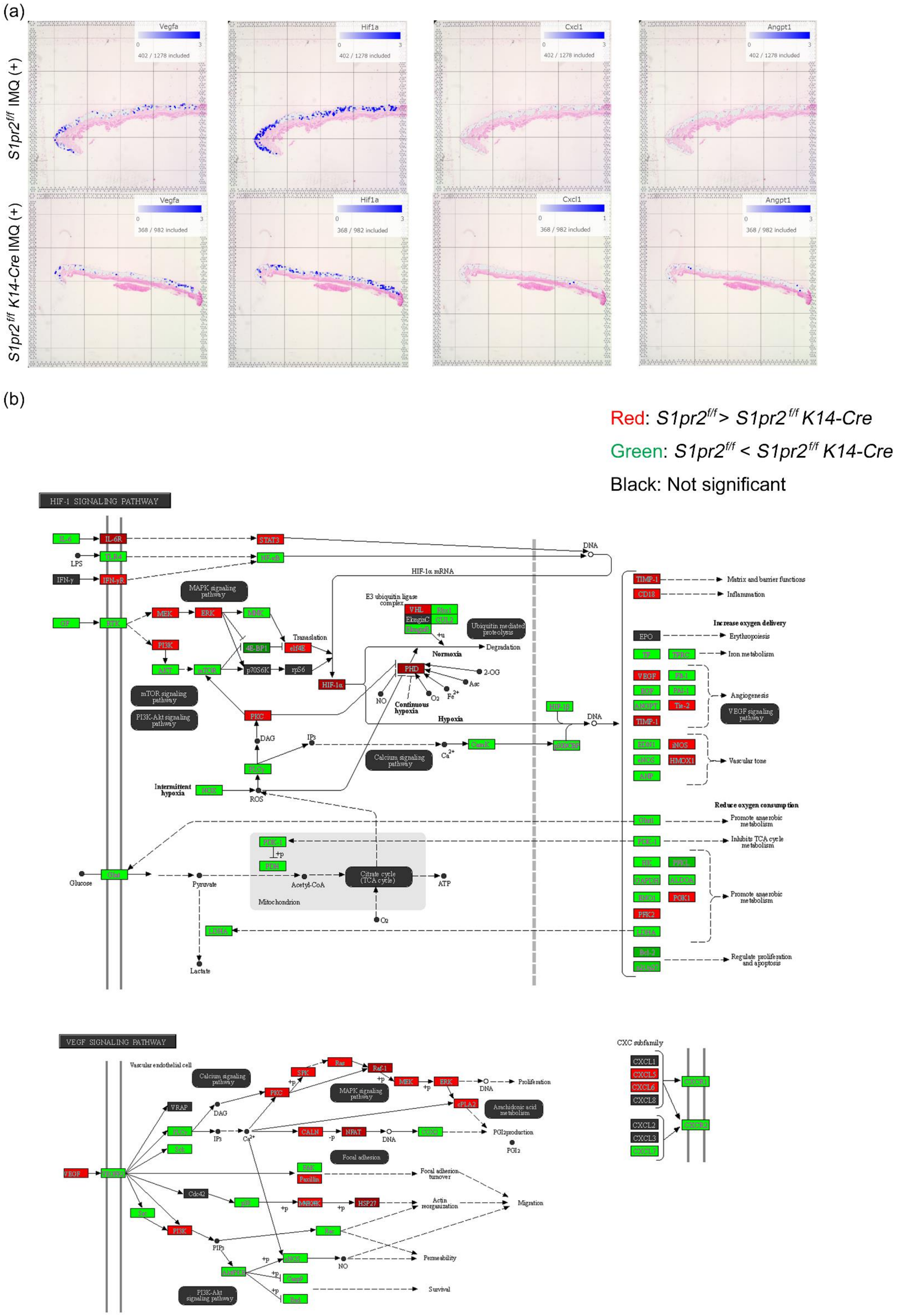

Supplementary Figure S4. S1PR2 deletion does not alter angiogenesis of psoriasis

(a) Gene expression levels of *Vegfa*, *Hif1a*, *Cxcl1*, and *Angpt1* analyzed by spatial sequencing.

(b) Pathway enrichment analysis of HIF-1 signaling pathway (upper panel) and VEGF signaling pathway (lower panel). *Vegfa*, vascular endothelial growth factor*;* *Hif1a*, hypoxia-inducible factor 1-alpha*;* *Cxcl1*, C-X-C motif chemokine ligand 1*;* *Angpt1*, Angiopoietin 1.
